# Trypsin-Catalyzed Deltamethrin Degradation

**DOI:** 10.1371/journal.pone.0089517

**Published:** 2014-03-03

**Authors:** Chunrong Xiong, Fujin Fang, Lin Chen, Qinggui Yang, Ji He, Dan Zhou, Bo Shen, Lei Ma, Yan Sun, Donghui Zhang, Changliang Zhu

**Affiliations:** 1 Department of Pathogen Biology, Nanjing Medical University, Nanjing, Jiangsu, China; 2 Jiangsu Province Key Laboratory of Modern Pathogen Biology, Nanjing, Jiangsu, China; IIT Research Institute, United States of America

## Abstract

To explore if trypsin could catalyze the degradation of non-protein molecule deltamethrin, we compared *in vitro* hydrolytic reactions of deltamethrin in the presence and absence of trypsin with ultraviolet-visible (UV/Vis) spectrophotometry and gas chromatography-mass spectrometry (GC/MS). In addition, acute oral toxicity of the degradation products was determined in Wistar rats. The results show that the absorption peak of deltamethrin is around 264 nm, while the absorption peaks of deltamethrin degradation products are around 250 nm and 296 nm. In our GC setting, the retention time of undegraded deltamethrin was 37.968 min, while those of deltamethrin degradation products were 15.289 min and 18.730 min. The LD_50_ of deltamethrin in Wistar rats is 55 mg/kg, while that of deltamethrin degradation products is 3358 mg/kg in female rats and 1045 mg/kg in male rates (61-fold and 19-fold reductions in toxicity), suggesting that trypsin could directly degrade deltamethrin, which significantly reduces the toxicity of deltamethrin. These results expand people's understanding of the functions of proteases and point to potential applications of trypsin as an attractive agent to control residual pesticides in the environment and on agricultural products.

## Introduction

Serine proteases are involved in diverse physiological and cellular processes, including food digestion, protein maturation, blood coagulation, host immune responses, fibrinolysis, tissue remodeling, fertilization, and embryogenesis [1]. Trypsin, a widely studied serine protease, is present in the digestive systems of vertebrates and invertebrates [2]. As the most abundant protease, its physiological importance, especially in vertebrate digestive functions, has been well documented [3]–[5]. In addition, recent works are pointing to possible novel functions of trypsin. In our previous study, trypsin gene was cloned from deltamethrin-resistant *Culex pipiens pallens* mosquito using a combination of suppression subtractive hybridization (SSH) and microarray. We found that trypsin gene was significantly up-regulated in deltamethrin-resistant *Culex* mosquito [6]. Stable transfection of trypsin gene into mosquito cells conferred protection against deltamethrin treatment. On the other hand, RNA interference-mediated down-regulation of trypsin in mosquito cells decreased deltamethrin resistance. These results suggest that trypsin might contribute to deltamethrin resistance in *Culex* mosquito [7]. Interestingly, in subsequent toxicity test of trypsin-treated deltamethrin in mosquitoes, our data suggest that trypsin may directly degrade deltamethrin. However, this possibility needs to be further tested.

To explore the potential function of trypsin, we investigated trypsin-catalyzed *in vitro* hydrolytic reactions of deltamethrin with ultraviolet-visible spectrophotometry (UV/Vis) and gas chromatography-mass spectrometry (GC/MS). In addition, acute oral toxicity of the degradation products in Wistar rats was measured.

## Materials and Methods

### Chemicals

#### Deltamethrin

[(S)-alpha-cyano-3-phenoxybenzyl-(1R,*cis*)-2,2-dimethyl-3-(2,2-dibromovinyl)-cyclopropanecarboxylate] was purchased from Roussel Uclaf Company (Paris, France, with more than 98% purity). Trypsin extracted from bovine pancreas and Nα-Benzoyl-L-arginine 4-nitroanilide hydrochloride (L-BAPNA) were purchased from Sigma Chemical Company (St. Louis, MO, USA). Other chemicals used in this study were of analytical grade.

### Animals

Wistar rats of both genders with body weight of 180–200 g were obtained from Shanghai Slac Laboratory Animal Co. [certificate no. SCXK (Hu) 2003–0003]. Animals were maintained in a barrier-sustained animal room with certified environmental condition [certificate no. SCXK (Su) 2002-0031]. Five rats of the same gender were kept in each cage, with unrestricted food and water supply until dosing procedure. The animal facility was maintained at a temperature of 22±1°C, with relative humidity of 50%±10%, and with a 12-h light/dark cycle. All the operation procedures of animals followed the NIH (US National Institutes of Health) guidelines and were approved by Nanjing Medical University Animal Care and Use Committee.

### Degradation of deltamethrin

Trypsin and L-BAPNA were handled according to the manufacturer's instruction. An optimal pH was determined by plotting trypsin activity on L-BAPNA against a range of pH from 5 to 11. For the degradation of deltamethrin catalyzed by trypsin, reaction products were detected using the method described by Leng and Gries [8]. The optimal pH in our reaction system was 8.0, which was in accordance with results reported before [9], [10]. Briefly, trypsin was dissolved in 50 mM Tris–HCl buffer (pH 8.0). One hundredth volume of 1 mg/mL deltamethrin stock solution in DMSO was added to trypsin solution and incubated at 37°C for 10 minutes. Remaining deltamethrin and its degradation products were extracted for the following measurements.

### Extraction

Degradation reaction mixtures were vortexed for 30 s upon the addition of 1 mL of acetone and 1 mL of cyclohexane for every 3 mL of reaction mixture, then centrifuged for 10 min at 2,000 rpm. The organic phase was transferred to a glass vial with screw top. The aqueous phase was then extracted twice with 1 mL of cyclohexane each time, and the organic phase was also transferred to a fresh glass vial. The organic phase was then combined and evaporated to dryness under a stream of nitrogen at room temperature. After completely drying, the residue was reconstituted in 200 µL of cyclohexane. A 1 µL sample was then analyzed by GC/MS [11].

### Ultraviolet-visible (UV/Vis) spectrophotometry

Spectrograms were obtained using a Cary 5000 UV/Vis spectrophotometer (Varian, Inc., USA) according to the method described by Modi and LaCourse [12]. The time course of deltamethrin degradation and kinetics experiments were carried out in 3-mL UV-transparent cuvettes using Cary WinUV Bio Package software (Version 3.0). Dissolved trypsin (1 mg/mL) was added into 23.34 µM deltamethrin and incubated at 37°C using a thermostatic circulating water bath. The absorbance at 264 nm was detected every minute, and a trypsin internal standard was evaluated with the same procedure except in the absence of deltamethrin. For the kinetics experiment, trypsin solution (1 mg/mL) was dissolved in buffer as described above, and different amounts of trypsin were added to deltamethrin solution to make a total volume of 3 mL. The mixtures were incubated at 37°C, and the increase in absorbance from 200 nm to 500 nm was measured over a time period of 10 min. Standard curve of deltamethrin assayed by ultraviolet spectroscopy was constructed at the same time.

### Gas chromatography-mass spectrometry (GC/MS)

A Saturn 2200 GC/MS equipped with a CP-3800 GC and a split/splitless injector port (Varian, Inc., USA) was used in the experiment. The analytical column was a DB-5 [crosslinked (5% phenyl)-methylsiloxane, 30 m in length, 0.25 mm inner diameter, 0.25 µm film thickness] fitted with a retention gap (uncoated and deactivated, 1 m×0.25 mm i.d.) (Agilent Technologies, USA). The GC/MS was programmed to perform a 1.0 µL splitless injection. The injector port was set at 220°C. The temperature in column oven was as follows: initial temperature was kept at 40°C for 2 min; then the temperature was increased to 260°C at 8°C/min and maintained at 260°C for 20 min, and the total run time was 50 min. The flow of the carrier gas (helium) was maintained at 1.0 mL/min in constant flow mode. The MS was operated in full scan mode.

### Acute oral toxicity study

The acute oral toxicity study was conducted using the up-and-down procedure according to OECD (Organisation for Economic Co-operation and Development) Test Guideline 425. A designated statistical software Acute Oral Toxicity (Guideline 425) Statistical Program (AOT425statPgm, version 1.0, developed by Westat) was used to guide the dosing procedure. The assumed Sigma value was 0.5. The amount of deltamethrin was measured before degradation reaction. After degradation and extraction, the amount remaining in aqueous phase and the degree of reaction completion were checked by vaporization of an aliquot, reconstitution in acetone and measurement by GC. We found negligible amount of deltamethrin and degradation products in aqueous phase, indicating their complete extraction into organic phase. The mixture of deltamethrin degradation products and unconverted deltamethrin from degradation reaction was reconstituted in corn oil and administered by gavage with a gastric feeding tube. The rats were fasted overnight before dosing. The dose progression followed the standard of OECD Guideline 425. After dosing, each rat was carefully observed for the first 5 min to make sure no fluid leaked out from the stomach. For short-term outcomes, rats were checked every 15 min for the first 4 h, every 30 min for the next 6 h, then every 6 h for the following 38 h. Animals surviving the first 48 h were observed for another 12 days with regular check every 6 h for long-term outcome. Any signs of intoxication including changes in skin and fur, eyes, mucosae were carefully observed. Behavioral and somatomotor manifestations of acute oral toxicity were also observed, including tremors, convulsions, short breath, salivation, diarrhea, lethargy, sleep and coma. Death was used as an endpoint, because the difference in lifespan provides important information about toxicity. All observations for each rat were systematically recorded. The LD_50_ were calculated by Maximum Likelihood Estimation (MLE).

## Results

### Deltamethrin degradation reactions catalyzed by trypsin

**Figure 1:**
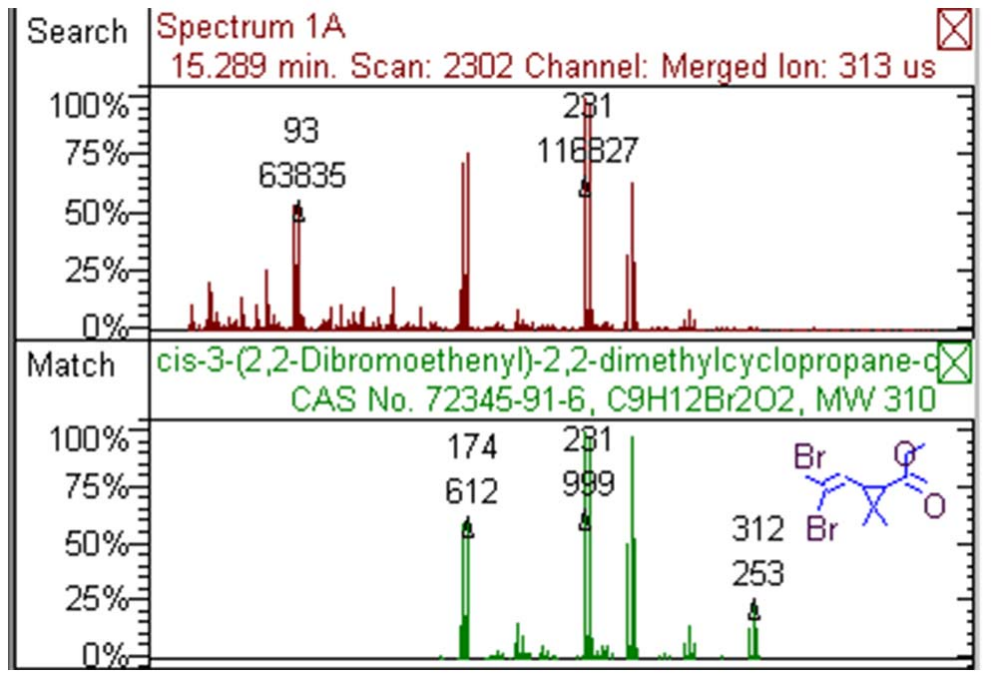
One of the main deltamethrin degradation products with the molecular formula of C_9_H_12_Br_2_O_2_.

**Figure 2:**
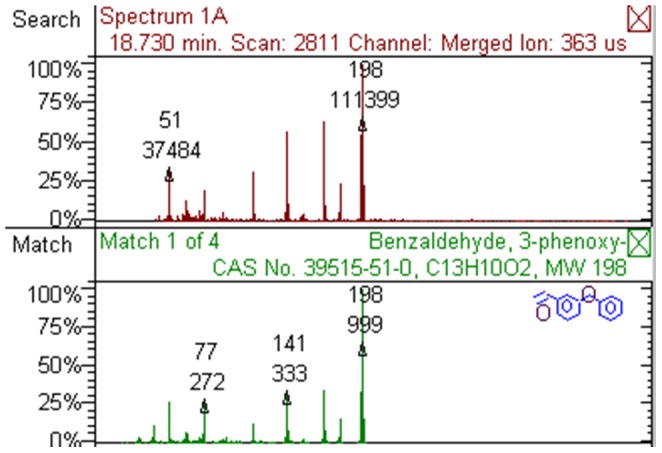
One of the main deltamethrin degradation products with the molecular formula of C_13_H_10_O_2_.

Both UV/Vis and GC/MS were employed to measure deltamethrin degradation products. In the UV/Vis test, the obtained spectra of deltamethrin before and after reaction were different, indicating at least some deltamethrin was converted to its degradation products. When the reaction mixture was subjected to GC/MS analysis, the retention times of crude products of deltamethrin degradation reaction (including undegraded deltamethrin) were 37.968 min, 15.289 min, and 18.730 min. Comparing the MS spectra with National Institute of Standards and Technology (NIST) Mass Spectral Database, it was found that the peak with 37.968 min retention time represented undegraded deltamethrin, and the other two represented degradation products of deltamethrin with molecular formula of C_9_H_12_Br_2_O_2_ and C_13_H_10_O_2_ (Fig. 1 and Fig. 2). The degrees of reaction completion were determined by GC as the ratios of converted deltamethrin to initial amount of deltamethrin, i.e. 1−(GC peak area of deltamethrin from a reaction)/(GC peak area of deltamethrin from unreacted control). At a trypsin concentration of 10 mg/mL, 94.9% deltamethrin was converted; at a trypsin concentration of 20 mg/mL, 97.7% deltamethrin was converted in our experimental setting (Table 1).

**Table 1:** Percentage of deltamethrin degraded in trypsin-catalyzed reactions.

Trypsin concentration (mg/mL)	0	1.25	2.5	5	10	20
GC peak area	525867	270343	111366	48176	27196	12475
Amount of unconverted deltamethrin (µg)	20	10.28	4.23	1.83	1.03	0.47
Degree of reaction completion (%)	0	48.6	78.9	90.9	94.9	97.7

### Acute oral toxicity study

The short- and long-term outcomes of the main test of deltamethrin and its degradation mixture are shown in Table 2. According to GC/MS results, a control group dosed with the amount of deltamethrin equal to the undegraded deltamethrin in the degradation reactions was also included. The toxicity symptoms included hyperspasmia, diaphoresis, salivation, and myasthenia in the posterior limbs. The onset of the symptoms was observed 2–3 h after administration, and the surviving rats recovered in 2–3 days. The LD_50_ of deltamethrin was 55 mg/kg in both male and female Wistar rats, while the observed LD_50_ of deltamethrin reaction mixture in male and female rats were 550 and 838.9 mg/kg, respectively (Table 2). After a correction for residual 5% deltamethrin in the reaction mixture according to the equation (1), the LD_50_ of deltamethrin degradation products were 1045 mg/kg and 3358 mg/kg in male and female rats, respectively. The acute oral toxicity of the degradation products was significantly lower than that of deltamethrin.
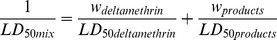
(1)Where* w_i_* is the mass fraction of substance *i.*

**Table 2:** The survival and death of Wistar rats after treatment of deltamethrin or its degradation products.

Group	Dosage (mg/kg) and outcome						LD_50_
Male rats treated with deltamethrin	55 (×)	17.5 (√)	55 (×)	17.5 (√)	55 (√)	175 (×)	55
Female rats treated with deltamethrin	55 (√)	175 (×)	55 (×)	17.5 (√)	55 (×)	17.5 (√)	55
Male rats treated with degradation mixture	175 (√)	550 (√)	2000 (×)	550 (√)	2000 (×)	_550 (_×_)_	550
Female rats treated with degradation mixture	175 (×)	55 (√)	175 (√)	550 (√)	2000 (×)	550 (√)	838.9

√ survival, × death.

### Pathological results

**Figure 3:**
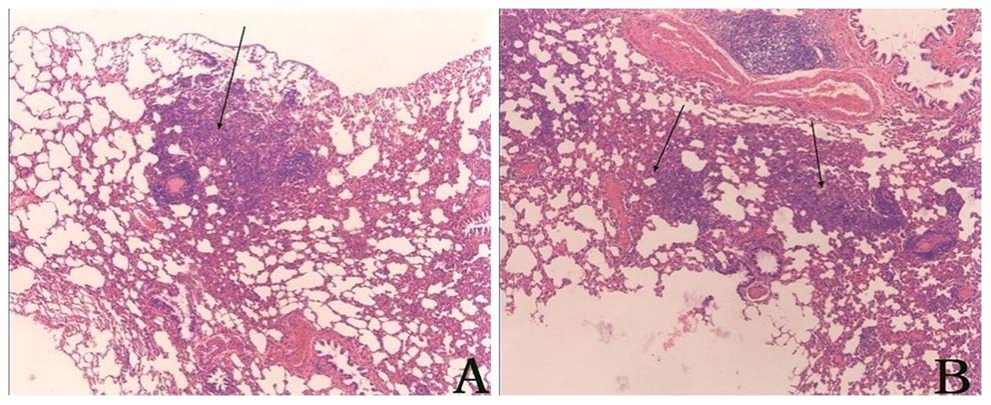
Acute toxicity-associated pathological changes in Wistar rats manifested as atelectasis. A) The group receiving deltamethrin degradation mixture; B) The control group receiving residual deltamethrin.

Because trypsin-catalyzed deltamethrin degradation mixture still contained 5% unconverted deltamethrin, a control group was administered with deltamethrin equal to the residual amount in the degradation mixture. No overt pathological change was observed in the nervous, cardiovascular, gastrointestinal and genitourinary systems. The skin, fur and mucosal surfaces also appeared normal. The animals did not display abnormal behaviors. During the acute phase, pathological changes were observed in the lung in the group administered with deltamethrin degradation products and the residual deltamethrin control group, with the group administered with degradation products showing more severe pathological changes. The damages occurred in the lung mainly manifested as atelectasis (Fig. 3).

**Figure 4:**
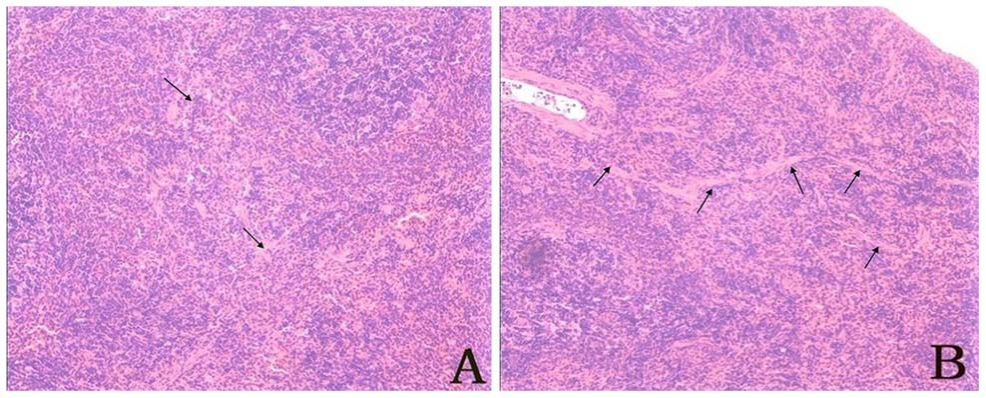
Delayed toxicity-associated pathological changes occurred mainly in the spleen, including fibrosis and lymphoid tissue hyperplasia. A) The group receiving deltamethrin degradation mixture; B) The control group receiving residual deltamethrin.

Delayed toxicity-related pathological changes mainly occurred in the spleen as fibrosis and lymphoid tissue hyperplasia. There was no significant difference between the degradation products group and the residual deltamethrin control group (Fig. 4).

## Discussion

In mammals, deltamethrin is subject to hydrolysis catalyzed by esterases such as carboxylesterases, with the main products being (1R,3R)-3-(2,2-dibromoethenyl)-2,2-dimethyl-cyclopropanecarboxylic acid and 3-phenoxybenzoic acid [13]. Similar to carboxylesterases, trypsin has a catalytic triad composed of serine, histidine and aspartate, suggesting that trypsin may be able to catalyze the breakdown of the single ester bond in deltamethrin since many proteases can catalyze the hydrolysis of both amide bonds and ester bonds [14]–[16]. Using UV/Vis spectrum analysis and GC/MS chromatograms, we found that deltamethrin could be degraded by trypsin *in vitro*. Two major degradation products were identified by matching the MS spectra with the MS database of NIST. One product with the molecular formula of C_9_H_12_Br_2_O_2_ was predicted to be (1R,3R)-rel-3-(2,2-dibromoethenyl)-2,2-dimethyl-cyclopropanecarboxylic acid, methyl ester (CAS number 72345-91-6). Considering the molecular configuration of deltamethrin, this product is likely (1R,3R)-3-(2,2-dibromoethenyl)-2,2-dimethyl-cyclopropanecarboxylic acid, methyl ester (CAS number 61775-87-9). The other product with the molecular formula of C_13_H_10_O_2_ was predicted to be 3-phenoxybenzaldehyde (CAS number 39515-51-0). These products were similar to what would be expected from ester bond hydrolysis. However, instead of (1R,3R)-3-(2,2-dibromoethenyl)-2,2-dimethyl-cyclopropanecarboxylic acid, its methyl ester was generated in the trypsin-catalyzed reaction. The origin of the methyl group is currently unknown, since no artificial methyl esterization step was included in the reaction. Alternatively, the degradation product may not be a methyl ester, but may be resulted from a substitution reaction of the carboxyl group in which the hydroxyl group was replaced by a nitrile group (possibly coming from the other breakdown product) which was subsequently hydrolyzed into an aldehyde group, i.e. carboxyl group converted into glycoaldehyde group. As about the other product, a possible reason that 3-phenoxybenzaldehyde was not converted into 3-phenoxybenzoic acid could be a lack of participation of oxidative enzymes that would be present *in vivo*. These results suggest that trypsin-catalyzed deltamethrin degradation is more complex than promiscuous esterase activity of trypsin. More detailed mass spectrometry studies using isotope-labeled deltamethrin may provide further information about the underlying mechanism.

Because the reaction is complex, and some minor products may be generated, we used the reaction mixture to test the change in toxicity instead of purifying two major products and testing them separately or in combination. Since the degradation mixture contained unconverted deltamethrin, the observed toxicity also included that of residual deltamethrin. The LD_50_ of degradation mixture was significantly higher than that of deltamethrin, indicating the products of trypsin-catalyzed deltamethrin degradation had significantly reduced toxicity in mammals than their parent molecule. Assuming the degradation products and deltamethrin assert their toxic effect in mammals with similar modes of action, in which case their toxicity could be arithmetically additive, we used the equation (1) to calculate the toxicity of the products. The validity of such correction is contingent to the nature of toxicity of the products. Nevertheless, qualitative comparison of their toxicities is always valid.

In the pathology study, to make sure the observed toxicity reflected the effect of the products, a control group administered with residual amount of deltamethrin was included. During acute phase, only pathological changes in the lung were observed. The group administered with degradation mixture displayed more severe damages than the residual deltamethrin group. These results suggest that both deltamethrin and its degradation products are toxic to the lung. One concern is that the damages in the lung were caused by fluid leakage during gavage, i.e. deltamethrin and its degradation products were directly administered into the lung instead of being delivered to the lung by circulation. In that case, our observation would still support the conclusion that deltamethrin and its degradation products are both toxic to the lung (when inhaled). The differences in damage were unlikely caused by different amounts of fluid leaking into the lungs because all animals in the degradation mixture group had more severe damages compared to residual deltamethrin group. On the other hand, because the rats receiving a full dose of undegraded deltamethrin displayed much more severe damages than those receiving degradation mixture, the degradation products were less toxic than deltamethrin.

Deltamethrin degradation products have much lower toxicity than deltamethrin as measured by both acute and delayed pathological damages. These results indicate that trypsin can directly degrade more non-protein molecules (organic pesticides) than previously realized. This will expand people's understanding of the functional properties of proteases.

Excessive pesticide residues in agricultural produces and environment pose a threat to human health. The fact that many pests are increasingly resistant to pesticides also aggravates the situation [17]. It is important to develop safe and effective methods to degrade pesticides as part of pollution control. Our data demonstrate that trypsin can directly degrade deltamethrin *in vitro* and significantly reduce its mammalian toxicity, suggesting that trypsin and possibly other proteases could be exploited in pesticide decontamination. Compared to some enzymes and bacterial strains that have been explored as potential tools for environmental bioremediation [18]–[20], trypsin has a unique advantage as it is unstable in aqueous environment around neutral pH due to its self-degradation. Our work suggests that trypsin could be selected as a promising candidate for biocatalyst to control pesticide pollution without causing secondary contamination. Alternatively, engineered microbes that secrete active trypsin may be used to control pesticide pollution in the environment.
